# Veterinarians’ brief guide to confidence intervals, standardized effect size, and number needed to treat: understanding the impact of treatments and disease

**DOI:** 10.3389/fvets.2026.1760216

**Published:** 2026-03-20

**Authors:** Brenna R. Pugliese, Kimberly L. Hallowell, Aditi Vigneshwar, Earl G. Ford IV, Shannon S. Connard, Kim Love, B. Duncan X. Lascelles

**Affiliations:** 1 Comparative Biomedical Sciences Graduate Program, College of Veterinary Medicine, North Carolina State University, Raleigh, NC, United States; 2 Translational Research in Pain, Department of Clinical Sciences, College of Veterinary Medicine, North Carolina State University, Raleigh, NC, United States; 3 Department of Molecular and Biomedical Sciences, College of Veterinary Medicine, North Carolina State University, Raleigh, NC, United States; 4 K. R. Love Quantitative Consulting and Collaboration, Athens, GA, United States; 5 Comparative Pain Research and Education Center, North Carolina State University, Raleigh, NC, United States; 6 Thurston Arthritis Center, UNC School of Medicine, Chapel Hill, NC, United States; 7 Center for Translational Pain Research, Department of Anesthesiology, Duke University, Durham, NC, United States

**Keywords:** confidence interval, effect size, number needed to treat, *p*-value, statistics

## Abstract

Veterinarians in all settings strive to practice evidence-based medicine, but current methods of reporting scientific study results create barriers to clinical translation of findings. The *p*-value is the most commonly used measure of statistical significance, but fails to convey information about clinical relevance of findings. In this article, the limitations of the *p*-value as a standalone measure and the importance of reporting additional metrics such as confidence intervals (CI), standardized effect size (ES), and number needed to treat (NNT) in veterinary research are discussed. Confidence intervals and ES can help describe the magnitude of an observed effect, whereas NNT is a practical estimate of the average number of animals that need to receive a treatment for one additional animal to experience ‘success’. Using examples of clinical studies on the effect of osteochondrosis on racing performance, silver nanoparticles for equid wound healing, and bedinvetmab for treatment of canine osteoarthritis, this article demonstrates the importance of reporting CIs, ES, and NNT in addition to *p*-values for providing deeper insights on treatment-impact and clinical-decision making. Incorporating the use of these tools in addition to the *p*-value in veterinary manuscripts will aid practitioners in making informed, patient-centered decisions.

## Introduction

In veterinary medicine, evaluating the effectiveness of treatments, interventions, or therapies is essential. As veterinary medicine has progressed over the years, there has become a stronger push for the practice of evidence-based medicine. The number of published research studies can be daunting, however, and the translation of research trial findings into information that is readily usable in the clinic is convoluted. *p*-values are commonly used in published veterinary studies to assess whether the results are statistically significant (typically, *p* < 0.05). However, they can be misleading:

1. A *p*-value does not indicate how large an effect is, or whether this effect is clinically meaningful (1). It only tells us how likely a sample is to produce a measured effect if firstly, the null hypothesis of the study is true (usually, that there is no underlying effect at all) (2) and secondly, the assumptions of the analysis are met.
2. A small *p*-value (e.g., *p* < 0.05) indicates statistical significance, but the clinical effect might be very small. Large sample sizes can result in a very small difference between group responses being labeled ‘significant’ (3).
3. Conversely, a large *p*-value (non-statistically significant) does not prove that there is no effect—it just means the evidence for an effect is weak or may be a result of a small sample size (3).

For example, imagine a hypothetical study on a new analgesic for post-operative pain relief in a population of 5,000 cats at a tertiary referral center. The objective of this fictitious study was to determine if a new opioid medication, PainFree-Cat, administered to cats following exploratory laparotomy is more effective at controlling post-operative pain than the current opioid treatment, buprenorphine. The Glasgow composite measure pain scale (CMPS) (4) for acute pain in cats was used (maximum score of 20) as the primary outcome measure. The null hypothesis was that there is no difference in mean CMPS scores at 4-h after surgery in cats receiving subcutaneous PainFree-Cat (*n* = 2,500) compared to cats receiving subcutaneous buprenorphine (*n* = 2,500).

If, when comparing groups (new treatment [PainFree-Cat] versus standard treatment [buprenorphine]) using a relevant measure [CMPS score], at a relevant time point [4-h], the study groups differed (in favor of the new treatment, PainFree-Cat) with a *p*-value of 0.04, this would mean new treatment is statistically significantly better than the comparator, buprenorphine, at the 5% level. But if the ‘effect’ is very small (say, only a 1-point improvement over standard on a 20-point pain scale), instigating the new treatment into clinical practice may not be worthwhile. The large sample size in this example, 2,500 cats per group, resulting in a small difference in pain score being labeled as significant, illustrates how *p*-values can be misleading (argument #2 in the above list). On the other hand, if the ‘effect’ was larger (say, 3 points), this would make a non-significant *p*-value of 0.06 clinically relevant, even though it is not ‘statistically significant’. The *p*-value is merely a measure of the likelihood of the observed data occurring under the null hypothesis. It cannot tell us whether the study hypothesis is true, nor how relevant study results are to clinical patients.

So, if *p*-values do not tell us about the degree of efficacy we are likely to see, how do we assess potential clinical utility? Confidence intervals (CIs) can improve the applicability of some of these statistical measures, giving readers an idea of both the magnitude of the effect and the degree of uncertainty in that magnitude. Confidence intervals are estimates which provide an upper and lower threshold to the estimate of the magnitude of effect and are conventionally reported as 95% CIs (5). Practically, this represents the range in which we, with 95% confidence, can expect a treatment effect to fall within (5). In this way, CIs help to overcome some of the limitations of *p*-values, by assessing a range of potential magnitudes of the treatment effect in practice. The reader must still be able to determine for themselves if the magnitude of change is clinically relevant for their patient population.

Standardized effect size (ES) measurements also provide a metric for how large the effect of a treatment is independent of sample size, increasing clinical ability beyond that of a *p*-value. Although categorizations of ES statistics exist for small, medium, and large effects, the categories are arbitrary and practitioners must again determine for themselves the clinical relevance of the ES. Finally, practical ES measurements such as number needed to treat (NNT) provide values that are directly translatable from research to clinical practice. These measures are uncommonly reported, however, and are only applicable to studies with dichotomous outcomes.

Here we discuss in detail the potential utility of confidence intervals, ES measurements, and NNT in veterinary research. By incorporating these tools into manuscripts, researchers can make their findings more accessible to practitioners, and the practice of evidence based medicine can be improved throughout the profession.

## Confidence intervals

Confidence intervals (CIs) provide a range of values for the magnitude of an effect within a given confidence, set by the investigator, but typically reported as 95% CIs (5, 6). A CI therefore sets the upper and lower threshold for the treatment effect that would be plausible within a given population (6). A 95% CI implies that if the study were carried out and treatment effect estimated 100 times, then 95 of the calculated intervals would be expected to contain the true value (6, 7). For clinicians, CIs help interpret study findings by understanding the actual magnitude of the difference between treatment groups, information that an isolated *p*-value does not provide, and giving clinical relevance to the observed effect (5). Importantly, CIs can be useful beyond examining treatment effects in a designed experiment: for example, CIs can also be used to describe disease effects, to report the magnitude of an effect relative to a disease state.

A confidence interval is calculated as: CI = point estimate ± margin of error, where point estimate is the sample mean calculated from the data and margin of error is the critical value appropriate to the distribution of the outcome, multiplied by the standard error of the point estimate. For analyses based on normal distributions (or approximations to normal distributions), the critical value is a z value derived from the normal distribution of the standard normal curve. Critical (z) values used in the calculation are 1.65, 1.96, and 2.58, respectively, for confidence levels of 90, 95, and 99%. In cases of numeric outcomes, where the point estimate is a sample mean, the standard error of the mean (SEM) is calculated as: SEM = Standard deviation/ $\sqrt{n}$ . In this situation, a 95% CI can therefore be simplified as: Sample mean ± 1.96 x (Standard deviation/ $\sqrt{n}$ ). As a logic check, one can see that the margin of error will be larger if the study had a small sample size (*n*) or if the data is highly variable (i.e., a larger standard deviation), demonstrating that margin of error depends on the size and variability of the sample (7). Standard errors (and margins of error) for many distributions and point estimates (such as percentages, differences, etc.) similarly rely on sample size.

To illustrate interpretation of reported CIs, we will examine a recent report determining the influence of clinical osteochondrosis (OC) on short- and long-term racing performance of standardbred racehorses (8). This retrospective case–control study included standardbred racehorses from a single breeding farm (*n* = 2,711), with 382 horses affected by OC (arthroscopically confirmed lesions) and 2,329 nonaffected horses. Racing performance data was evaluated using multiple linear regression models and 95% CIs were calculated. The study reports that compared to nonaffected horses, long-term OC-affected horses had an average of 8.8 fewer starts (95% CI = −14.4 to −3.2, *p* = 0.002). To begin, this result can be interpreted as: the mean difference in race starts is statistically different between groups, since *p* < 0.05. However, this does not give the reader any insight into the magnitude of the effect. The reporting of CI in this example provides this context: the mean difference in race starts between OC-affected horses and nonaffected horses was 8.8, and we have 95% certainty that the true value (mean difference in race starts between the two groups in the population) is between 3.2 and 14.4. It is important to note here, that interpreting this CI clinically, one must have a knowledge of horse racing starts and what a relevant change in this metric is. The clinician must have sufficient background knowledge to consider the different scenarios at the upper and lower end of the CI. If an OC-affected horse has 3.2 fewer race starts than its counterpart, would this be a clinically important difference? Conversely, if a horse has 14.4 fewer race starts, how clinically important is this? When the clinical impact does not change when considering the upper and lower threshold of the CI, the clinician can be more certain that the disease state is impactful to the patient (in this case, that an OC diagnosis meaningfully alters the number of race starts). Conversely, if there is a clinically different impact when considering the upper and lower thresholds, the reader should be more reserved in their interpretation of the 95% CI and the effect of OC on race performance. Recall that the *p*-value of 0.002 implies that there is a difference in race starts between OC-affected and nonaffected horses; however, the *p*-value alone lacks clinical relevance since no details about the magnitude of effect are gleaned (5). In summary, the 95% CI can help clinicians more completely interpret scientific evidence, providing more scope than a *p*-value, but the requirement for sufficient background knowledge for interpretation may be considered a disadvantage of this statistical approach. Importantly, a CI can be reported in combination with a standardized ES (Cohen’s D) or a NNT statistic, which adds the context of the magnitude of a given effect to these point estimates.

## Standardized effect size

Effect size describes the magnitude of a difference between two groups (e.g., treatment vs. control) allowing the practitioner to interpret the potential clinical significance of a finding (3). Effect size quantifies how large or small the observed treatment effect is, independent of sample size. Effect size is important in understanding how impactful a treatment is, offering more information than a *p*-value.

One commonly reported standardized measure of ES in the human medical literature is Cohen’s d, particularly when comparing different treatments (9), and we are starting to see this useful metric reported in veterinary medicine (10, 11). Cohen’s d ES is calculated from the equation: d = (mean of treatment group – mean of control group)/pooled standard deviation. The resulting number gives a sense of how large the difference is between the two groups in terms of standard deviation units. Cohen suggested that d = 0.2 be considered a “small” ES, 0.5 represents a “medium” ES and 0.8 a “large” ES (12). Importantly, however, Cohen himself noted that these sizing conventions are relative to the area of research and research methods being used; he acknowledged the risk in creating “conventional operation definitions” that might be applied too broadly in diverse research fields.

**Figure 1:**
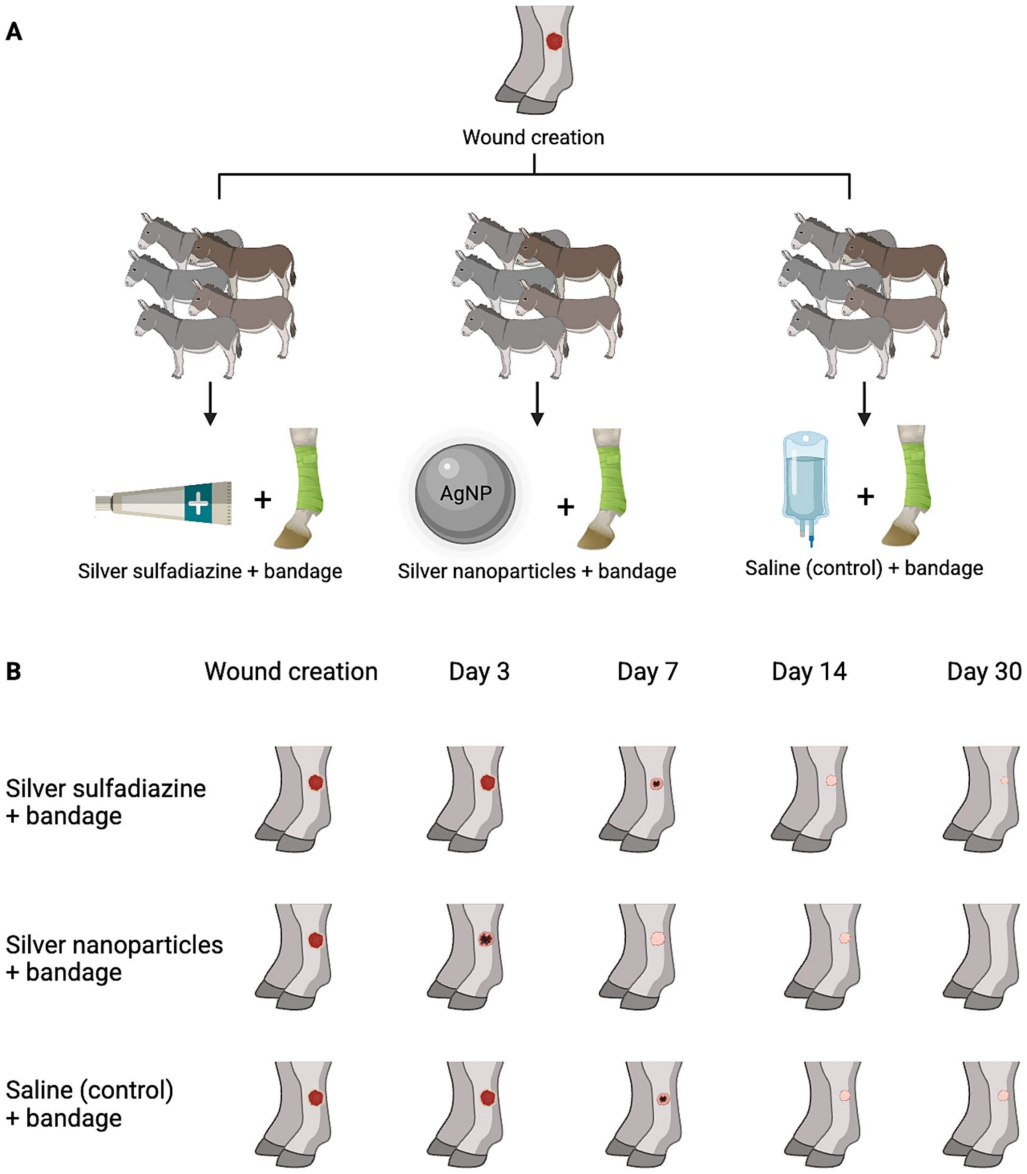
Evaluating clinical significance of silver nanoparticles (AgNPs) in wound therapy through standardized effect size. **(A)** Study design illustrating treatments compared in the investigation. **(B)** Progression of wound healing in donkeys treated with silver sulfadiazine and AgNPs compared to control limbs treated with saline. All limbs were bandaged.

Consider this example of an article about the use of a silver nanoparticles (AgNPs) wound dressing to treat distal limb wounds in donkeys (13). All donkeys received standard-sized cutaneous wounds (medial and lateral) on both metacarpal and metatarsal regions. Animals were kept on stall rest, and had bandages changed daily on both medial and lateral wounds. Group A received silver sulfadiazine (SSD), Group B received AgNPs, and Group C served as a control with no saline treatment (Figure 1). The rate of wound epithelialization was measured by calculating the surface area of the wounds over time (cm^2^/day). The study found statistically significant differences between all groups (*p* < 0.05), with the epithelialization rates being 0.11 cm^2^/day for the control, 0.12 cm^2^/day for the SSD group, and 0.15 cm^2^/day for the AgNPs group. Based on statistical analysis, the authors concluded that AgNPs was superior to SSD because it was associated with a statistically significant increase in epithelialization rate. However, statistical significance alone does not confirm clinical relevance. Here, the practitioner should ask themselves, “is it of *clinical significance* that my patient’s wound epithelializes at a rate that is 0.03 cm^2^/day faster with AgNPs? Does it change case management, patient outcome, or timeline for healing?” In this case, the answers to all of these questions are yes. When we calculate the ES for AgNPs vs. SSD we get a Cohen’s d of ~1.90 which is considered to be a large ES. This large ES supports the clinical interpretation that, even though daily differences in epithelialization may not be evident, a wound of this size in this patient population (donkeys) could experience clinically relevant outcomes from slight improvements in healing rate. An increase of just a few millimeters per day can reduce recovery time by approximately one week. This translates to fewer bandage changes, less intensive wound care, shorter stall rest, and reduced risk of complications, making AgNPs not just statistically superior, but also a clinically impactful choice. While the difference between the SSD and AgNPs groups was statistically significant, it is essential to evaluate its clinical relevance using Cohen’s d. While statistical significance indicates a difference is unlikely due to chance, ES conveys the magnitude and practical impact, helping assess clinical relevance.

Cohen’s d is descriptive and does not convey the precision of an estimate. The accuracy of it is influenced by small sample sizes, unequal variances, or high intra-group variability. Cohen cautioned that his benchmarks of small, medium and large ES should “not be applied mindlessly”. Hence, practitioners should interpret the clinical relevance based on the biological context of the investigation. Therefore, for a better understanding of treatment effects, investigators should consider reporting the raw mean differences, standard deviations, sample sizes, and confidence intervals along with Cohen’s d.

## Number needed to treat (NNT)

Number needed to treat (NNT) is a measure of treatment efficacy that can be readily applied to clinical patients (9). Number needed to treat is a statistic that helps determine the average number of animals that need to be treated with a specific intervention to prevent one bad outcome – which, when evaluating efficacy of a therapeutic, can be interpreted as the average number of animals that need to be treated with a specific intervention for one animal to benefit from the treatment, compared to a control group. In simple terms, it answers the question: “How many animals need to receive treatment to expect one additional positive outcome?” In this context, one has to remember that ‘benefit’, or ‘positive outcome’ means the individual animal has to improve by a *set amount*. Ideally, this is by an amount deemed to be clinically meaningful, the definition of which is discussed further below. Number needed to treat values can only be calculated when outcomes are dichotomized as success versus failure.

**Figure 2:**
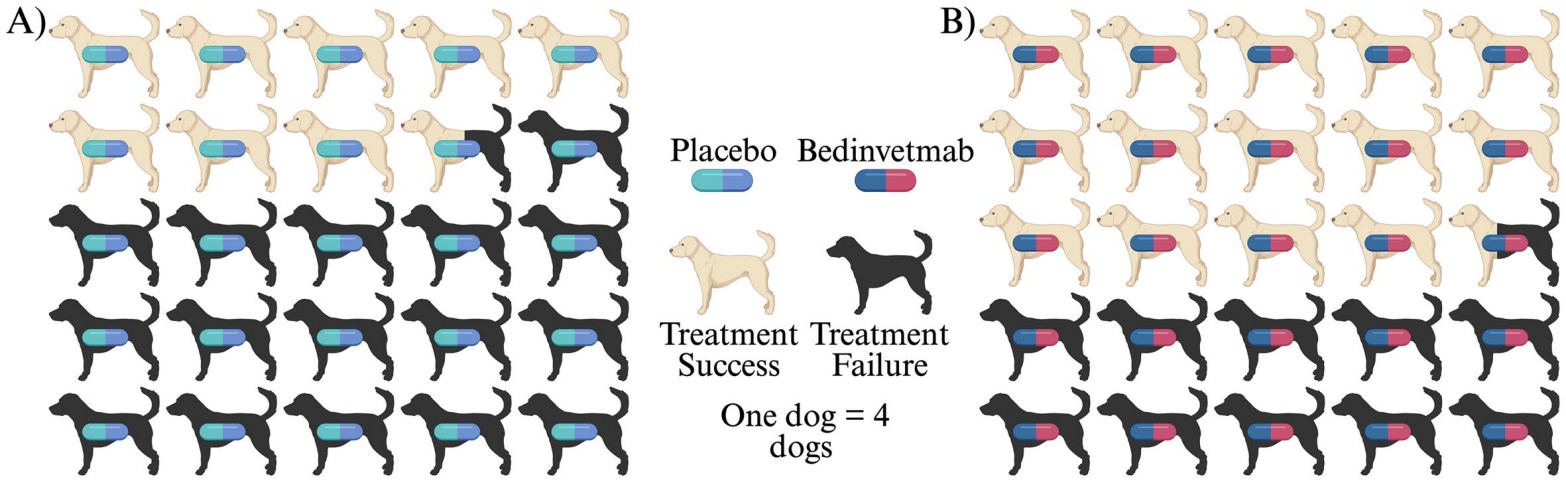
Evaluating efficacy of a canine osteoarthritis treatment using number needed to treat. **(A)** Placebo treated dogs showed a treatment success rate of 34.2%. **(B)** Bedinvetmab treated dogs showed a treatment success rate of 57.4%. Number needed to treat calculated at 4.3 from the equation: (1/EER-CER) (100).

Number needed to treat for a particular treatment is related to the Absolute Risk Reduction (ARR) which is calculated from the following: ARR = EER - CER, where EER = Experimental Event Rate and CER = Control Event Rate. We can then find NNT by the following: NNT = 100%/ARR. We can use a recent study evaluating treatment efficacy of the novel osteoarthritis pain management drug bedinvetmab, an anti-nerve growth factor (anti-NGF) monoclonal antibody, as an example (14):

At day 84 of treatment with either bedinvetmab or placebo, the bedinvetmab group had a treatment success rate of 57.4% (this is our EER or rate of success with bedinvetmab), while the placebo group had a treatment success rate of 34.2% (this is our CER or rate of success with the control) (Figure 2). If we subtract the placebo success rate from the bedinvetmab success rate we get our absolute risk reduction, 23.2%. Clinically it is hard to decide what this 23.2% means. However, by converting ARR to NNT (NNT = 100/ARR) we can more easily understand the impact of bedinvetmab. With this, we find an NNT of 4.3, meaning for every 4.3 patients treated, we expect that one will have *significantly reduced pain* due to the treatment with bedinvetmab.

At this point, it is important to understand what *significantly reduced pain* means. Number needed to treat tells us how many animals reach a pre-defined threshold. In the context of the study described above, the threshold for improvement was set based on improvement by a certain amount in both parts of a validated owner questionnaire, the Canine Brief Pain Inventory (CBPI) (15). The threshold was derived from work performed to determine the change in scores that would best differentiate treatment with an NSAID from a placebo, and therefore represents a clinically important change (16). In veterinary medicine pain management, we are just starting to see work being performed for different measures to determine the minimally clinically important difference (MCID) (17). Defining what a clinically meaningful change is across various measurement tools is essential to optimize the definition of ‘success’, and therefore the interpretation of NNT values.

When looking at treatment efficacy, a lower NNT is better. But another question clinicians should ask is “what is a ‘good’ NNT for this type of disease or treatment?” To evaluate a new treatment, the clinician needs to know the NNT values for similar treatments, or treatments used for the same condition. Unfortunately, there are few reported NNT values in the veterinary literature, and often NNT values need to be calculated from source data. Going back to the example above, we can ask “what are expected NNT values for the established treatment modality, NSAIDs?” The NNT for NSAIDs in humans is between 3 and 13 depending on the criteria for success (10). The NNT values for dogs treated with NSAIDs are between 4 and 8 using publicly available source data to calculate NNTs (18–20). Using these numbers as a frame of reference, we can see that the calculated NNT of 4.3 for bedinvetmab is similar to the NNT values for previously established therapies.

The final question a practitioner should ask related to NNT is “how similar is my patient to the study population?” Number needed to treat values reported in (or calculated from) a study pertain to the type of animal recruited to the study. In general, NNT will be lower if the patient being treated in practice has more severe disease (in this case, more severe pain) than in the patients in the study population, and the NNT will be higher with less severe disease. Clear reporting by researchers of how study populations were selected is thus critical to enable others to apply results to their practice population.

## Summary

In summary, *p*-values are insufficient on their own for drawing serviceable conclusions from a study because they do not tell us about the clinical significance or size of the effect. They inform us of the likelihood that an effect as large as the one seen in our sample would occur without an underlying effect in the population, but not how important that effect might be in a real-world context. Confidence intervals aid in the understanding of unstandardized effect sizes by quantifying the state of knowledge about what the actual effect may be, and with sufficient knowledge of the outcome scale can be helpful in the clarification of the meaningfulness of the finding. However, this is relatively unhelpful without a strong sense of what is an important difference in the outcome measurement. Both researchers and clinicians need to have an intuitive understanding of the outcome that they are using, including what magnitude of change is clinically relevant, to determine a useful study. The changes in each are based on the scales that are used and require a deep understanding of the changes necessary to be relevant in the particular clinical setting. Standardized ES, on the other hand, can suggest the magnitude of the treatment’s impact. Number needed to treat tells us how many animals we can expect to treat in order for one to be a treatment success (strictly, it tells you how many animals need to be treated on average to prevent one negative outcome – lack of success).

We encourage authors to report NNT and ES values as the data allow. The use of NNT and ES in combination allows a researcher or clinician the ability to better discern the importance of outcome even when the research is cutting edge. The concept of generalizability (also called external validity) of results is also critical: the idea that the results of a specific research study are useful for informing a clinical decision for patients presenting for care (21). We anticipate that with increased comfort in the interpretation of NNT and ES, rather than *p*-values and CIs alone, veterinary clinicians can increase their proficiency in the generalizability of research findings to treatment decisions in for own cases. Ultimately, the goal of the researcher should be to choose and utilize an appropriate outcome measurement that has practical value for the audience at hand. This will provide the best understanding of the information and allow for the best communication of their results in a clinically relevant way.

## Data Availability

The original contributions presented in the study are included in the article/supplementary material, further inquiries can be directed to the corresponding author.
